# Inflammatory and Anti-Inflammatory Response of the Ocular Surface After Excimer Laser Treatment

**DOI:** 10.3390/biomedicines14061338

**Published:** 2026-06-12

**Authors:** Mirko Resan, Andreas Kreis, Igor Pančevski, Željka Cvejić, Valentin Pajić, Bogdan Resan, Katarina Katanić-Pasovski, Dragana Ristić, Martina Kropp, Gabriele Thumann, Ivo Guber, Danilo Vojvodić, Bojan Pajić

**Affiliations:** 1 Faculty of Medicine of the Military Medical Academy, University of Defense, 11000 Belgrade, Serbia; katarinapasovski76@gmail.com (K.K.-P.); dadana25@yahoo.com (D.R.); vojvodic.danilo@gmail.com (D.V.); bojan.pajic@orasis.ch (B.P.); 2 Department of Physics, Faculty of Sciences, University of Novi Sad, 21000 Novi Sad, Serbia; zeljka.cvejic@df.uns.ac.rs; 3 Division of Ophthalmology, Department of Clinical Neurosciences, Geneva University Hospitals, 1205 Geneva, Switzerland; ajkreis@gmail.com (A.K.); martina.kropp@unige.ch (M.K.); gabriele.thumann@hug.ch (G.T.); ivo.guber@augenchirurgie.ch (I.G.); 4 Experimental Ophthalmology, University of Geneva, 1205 Geneva, Switzerland; 5 “Denica” Specialized Eye Hospital, 1000 Skopje, North Macedonia; dr.panchevski@gmail.com; 6 Medical Faculty, Semmelweis University, 1094 Budapest, Hungary; valentin.pajic@stud.semmelweis.hu; 7 Medical Faculty, University of Belgrade, 11000 Belgrade, Serbia; bogdanresan@yahoo.com; 8 Eye Clinic ORASIS, Swiss Eye Research Foundation, 5734 Reinach, Switzerland

**Keywords:** inflammation, cytokines, ocular surface, excimer laser, laser in situ keratomileusis (LASIK), photorefractive keratectomy (PRK), wound healing response

## Abstract

**Background/Objectives**: The goal of this study was to show the consistency between the inflammatory and anti-inflammatory response in the early postoperative period after corneal refractive surgery (LASIK and PRK), represented by the levels of specific cytokines in the tear film. **Materials/Methods**: A total of 68 myopic eyes (31 in the LASIK and 37 in the PRK group) had 3 samples of tear film taken, one before surgery (t_0_), another 1 h after surgery (t_1_) and the third 24 h after surgery (t_2_). The tear samples were then analyzed by flow cytometry in order to determine the levels of pro-inflammatory cytokines (IL-1β, IL-6, IL-8, IFN-γ, TNF-α) and the levels of anti-inflammatory cytokines (IL-4, IL-10). **Results**: A statistically significant positive correlation was found between the concentrations of all pro-inflammatory cytokines (IL-1β, IL-6, IL-8, IFN-γ, TNF-α) and all anti-inflammatory cytokines (IL-4, IL-10), respectively, for both t_1_ and t_2_ in the group of eyes that underwent LASIK. In the PRK group, there was a statistically significant positive correlation for all cytokines (pro-inflammatory and anti-inflammatory) at t_1_; however at t_2,_ there was no correlation between IL-1β and IL-10, IL-6 and IL-10, IL-8 and IL-10, and IFN-γ and IL-10. In contrast, for other correlations between pro-inflammatory and anti-inflammatory cytokines in the same observation time (t_2_), a positive correlation was observed. **Conclusions**: There is a consistency between the inflammatory and anti-inflammatory responses in the early postoperative period after refractive surgery, which persists longer after LASIK compared to PRK. This difference may explain the different clinical courses in patients after undergoing these procedures.

## 1. Introduction

The ocular surface has a specific anatomy and physiological mechanisms which help to regulate the immune response to prevent excessive and potentially harmful inflammation, caused by infection or injury, including surgical trauma. This reduction in the immune response is called ocular immune privilege. The consistency between the pro-inflammatory and anti-inflammatory reactions of the ocular surface is crucial for preserving corneal functional integrity [1].

The tear film, which covers the cornea and conjunctiva, has a complex structure and specific composition which protects the cornea, stimulates wound healing after injury, and maintains comfort [2]. The state of the tear film, its quality, and composition, can indicate certain systemic diseases [3]. The protein composition of the tear film is a reflection of ocular surface health. The tear film can be easily sampled and its composition can be analyzed by different methods. Specifically, the presence of different cytokines can be detected, including pro-inflammatory and anti-inflammatory cytokines. By detecting these cytokines, we can measure the immune response of the ocular surface, including the immune response after the surgical trauma of excimer laser treatment.

Laser in situ keratomileusis (LASIK) and photorefractive keratectomy (PRK) are leading corneal refractive surgical procedures across the world which still use an excimer laser. An excimer laser functions on the principle of photoablation on the corneal stroma, which remodels the curvature of the cornea and corrects existing refractive error. Apart from the correction of refractive errors, both procedures also lead to surgically induced trauma, which consequently results in an immune response of the complete ocular surface. For both procedures to be successful, which is most often the case, the aim is to preserve the transparency of the cornea as it is the main physiological and clinical characteristic of a healthy cornea. Corneal transparency is maintained thanks to the consistency between pro-inflammatory and anti-inflammatory reactions activated after the procedure. In this regard, the coherence of these reactions is very important. An excessive activity of the inflammatory response would lead to unwanted occurrences, such as the postoperative appearance of subepithelial haze after PRK [4] or diffuse lamellar keratitis after LASIK [5].

The recovery process of the ocular surface (cornea and conjunctiva) after trauma caused by photoablation of the corneal stroma is complex. Its mechanism and genetic control are still not well understood. This process includes an integrated function of numerous cytokines, growth factors, and proteases produced by corneal epithelial cells, stromal keratocytes, tear gland cells, and inflammatory cells. The protein profile in tears, including the profile of cytokines, changes dramatically after photoablation of the stroma. Known for their important role in this process are the following cytokines and growth factors: IL-1, IL-6, IL-8, TNF-α, PDGF-BB, TGF-β1, VEGF, HGF, and NGF [6,7]. The involvement of IL-1, IL-6, IL-8, and TNF-α and their excessive production have been proven in several studies [8,9,10,11]. The first stage of the stromal recovery process after photoablation is the removal of damaged tissue. Following this is the replacement of the damaged tissue with new tissue, and finally, the control and stopping of excessive growth and proliferation. The objective is to maintain normal physiology, and thus the transparency of the stroma [7]. Er and Uzmez in their study point out the significance of IL-6 in the recovery process of the corneal epithelium and state its importance in the stimulation of re-epithelization [12]. Weng et al. in their study point out the significance of IL-1β in the recovery process and state that this cytokine stimulates keratocytes to produce other cytokines [13]. West-Mays et al. in their study show that IL-1α is important in the recovery process because it leads to increased production of collagenase [14]. Malecaze et al. in their study point out the significance of IL-6 in increased collagen production and reduced production of metalloproteinases-2 (MMP-2) by keratocytes [9]. Cao et al. [15] assert the role of IL-1β and IL-10 in stimulating the apoptosis of stromal cells in the recovery process of the cornea after trauma caused by transepithelial excimer laser treatment. When it comes to modulation of the corneal response to trauma, Ellenberg et al. [16] in their study show that IL-1 and IL-8 are involved in this response by inducing angiogenesis, while Volpert et al. [17] state that IL-4 causes inhibition of angiogenesis, and Cao et al. [15] state that IL-1β and IL-10 stimulate the apoptosis of stromal cells.

The objective of our study is to examine the connection between refractive surgical procedures which use an excimer laser (LASIK and PRK) and the change in cytokine levels in tears in the early postoperative period, focusing in particular on the consistency between the pro-inflammatory and anti-inflammatory reactions which enables the preservation of corneal postoperative transparency, and thus ensures the effectiveness and safety of these procedures. We followed the pro-inflammatory reactions of the ocular surface through levels of pro-inflammatory cytokines (IL-1β, IL-6, IL-8, IFN-γ and TNF-α) as well as anti-inflammatory reactions through levels of anti-inflammatory cytokines (IL-4, IL-10) in tears in the early postoperative period (the first 24 h).

Pleiotropy is a feature of IL-6 because, apart from displaying pro-inflammatory effects, it also has regenerative and anti-inflammatory effects. In the literature, it is mostly known for its pro-inflammatory effect, and that is why we included this cytokine in the pro-inflammatory group [18].

## 2. Materials and Methods

We performed a prospective clinical cohort study. To perform the study, we obtained the approval of the Ethical Board of the Military Medical Academy in Belgrade. With the informed consent of each participant, 35 participants were included in total, with 68 myopic (short-sighted) eyes (2 participants had 1 eye operated). The refractive error of all eyes reached up to −3.0 spherical diopters. The eyes included in the study were divided into two groups, based on the performed surgical method:

1. LASIK group: Eyes treated with LASIK (*n* = 31);
2. PRK group: Eyes treated with PRK (*n* = 37).

Three samples of tears were taken from each operated eye—preoperative (t_0_), one-hour postoperative (t_1_), and twenty-four-hours postoperative (t_2_)—resulting in two hundred and four tear samples analyzed. The participants did not undergo any form of anti-inflammatory therapy before and 24 h after the intervention.

Each participant in the study had to be healthy, with no ocular or general disease. Preoperative evaluation included the following: ophthalmological and general medical history; automated refracto-keratometry; the best corrected visual acuity (BCVA); Schirmer test; anterior eye segment examination on slit lamp; corneal topography examination on Allegro-Oculyzer (Wavelight, Erlangen, Germany); Goldmann applanation tonometry and a dilated fundus examination.

LASIK and PRK were performed on a Wavelight Allegretto (400 Hz) excimer laser (Wavelight, Erlangen, Germany) with stromal photoablation in a 6.5 mm optical zone. By using this optical zone for all treatments, we minimize the variance in the energy used during the procedure [19]. During LASIK, we used a microkeratome (One Use-Plus SBK, Moria, France) to create a corneal flap with a thickness of 130 µm, and during PRK the corneal epithelium was removed by a rotational brush (Amoils, Innovative Excimer Solutions, Inc., Toronto, ON, Canada). Interventions were carried out in topical anesthesia with anesthetic eyedrops (oxybuprocaine 4 mg/mL). Since the drops of topical anesthetic were applied only once after preoperative tear sampling (t_0_), their toxic effect on the ocular surface [20] as well as their influence on cytokine production were excluded. At the end of each intervention (LASIK and PRK) antibiotic eyedrops without steroids (tobramycin 3 mg/mL) were applied in each treated eye. Soft therapeutic contact lenses (Ciba Vision Night and Day, Ciba Vision, Duluth, MN, USA) were placed on the eyes at the end of PRK, which were removed on the fifth postoperative day. One hour after the intervention, the second sample of tears was collected from each treated eye (t_1_). On the first postoperative day, all the participants were prescribed antibiotic eyedrops (tobramycin 3 mg/mL) four times per day and preservative-free artificial tears (sodium hyaluronate 1 mg/mL) every hour. So, the participants were without anti-inflammatory therapy on the first postoperative day, and 24 h after the intervention, the third sample of tears was collected from each treated eye (t_2_). After the third sampling of tears, the participants were given topical therapy according to the protocols for postoperative therapy in LASIK and PRK.

The tear samples in our study were collected using the method described in the study by Acera et al. [21]. The tear samples were taken from the lower lateral tear meniscus with minimal irritation of the ocular surface and the edge of the eyelid, without anesthetic. Each tear sample was collected using a cellulose microsurgical sponge (MicroSponge™ regular tip, Alcon, Inc., Hünenberg, Switzerland). After sampling, the tear fluid was separated by centrifuging the sponge in 0.5 mL of phosphate-buffered saline. The samples were centrifuged at 13,000 rpm for 15 min at 4 °C (MPW-350r; MED Instruments, Warszawa, Poland). The samples of tear liquid were kept at a temperature of −80 °C until the final analysis of cytokine levels.

The cytokine level in tear fluid was determined by flow cytometry using the Beckman Coulter FC 500 flow cytometer with CXP analysis software (Version 2.2) and a commercial test kit designed for the detection of cytokine complexes in small sample volumes: TNF-α, TNF-β, IL-1β, IL-2, IL-4, IL-5, IL-6, IL-8, IL-10, IL-12, IFN-γ (the Human Th1/Th2 11-plex FlowCytomix Multiplex, Bender MedSystem, Vienna, Austria). The level of cytokines was measured in pg/mL. In our study, the cytokines of interest were the pro-inflammatory cytokines IL-1β, IL-6, IL-8, IFN-γ, and TNF-α, as well as the anti-inflammatory cytokines IL-4 and IL-10.

For statistical data analysis, a Shapiro–Wilk test was first performed, showing that the data has a non-parametric distribution. Thus, Mann–Whitney-U test and Spearman’s correlation coefficient were used. From descriptive statistics, we calculated the mean and median of all results.

## 3. Results

The study included 35 participants, with 68 myopic (short-sighted) eyes (2 participants had 1 eye operated). A total of 16 participants were included in the LASIK group (9 males and 7 females), while the PRK group included 19 participants (14 males and 5 females). The average age of participants in the LASIK group was 34 (33.81 ± 6.52) yrs, and in the PRK group, it was 33 (33.05 ± 6.11) yrs.

**Figure 1:**
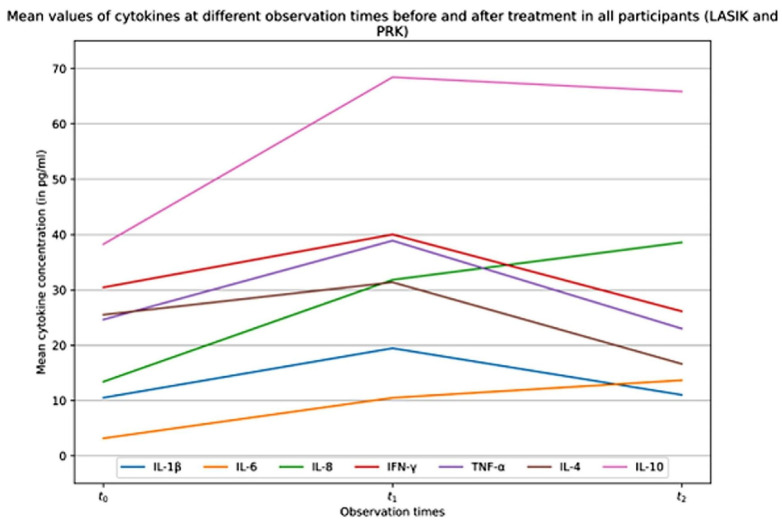
Cytokine levels (in pg/mL) at different observation times expressed through the mean value in all operated eyes (LASIK and PRK methods).

**Figure 2:**
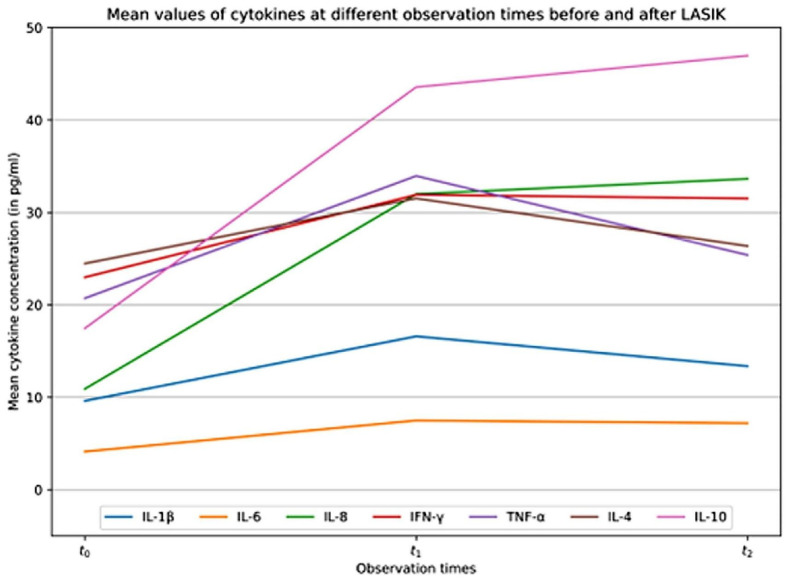
Cytokine levels (in pg/mL) at different observation times expressed through the mean values in eyes that underwent LASIK.

**Figure 3:**
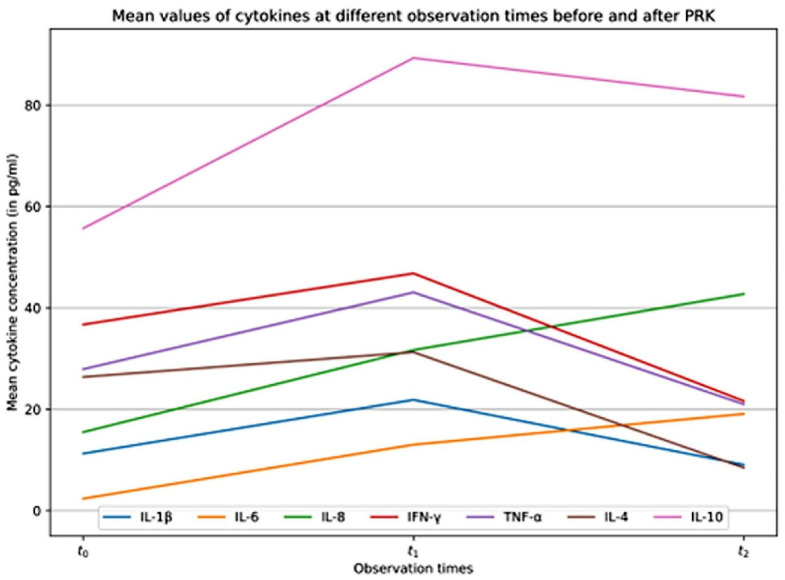
Cytokine levels (in pg/mL) at different observation times expressed through the mean value in eyes that underwent PRK.

The results of our study, with the statistical significance of the analyzed data, are presented in the following Table 1, Table 2, Table 3, Table 4, Table 5 and Table 6 and Figure 1, Figure 2 and Figure 3.

**Table 1:** Values of cytokines (in pg/mL) at different times of observation expressed through the mean and median values in all treated eyes (operated by LASIK and PRK methods).

All Participants	Tear Sampling Time					
Cytokine	t_0_		t_1_		t_2_	
	Mean	Median	Mean	Median	Mean	Median
IL-1β	10.508	0	19.461	2.470	11.023	0
IL-6	3.166	0	10.494	0	13.669	0
IL-8	13.404	0	31.828	39.970	38.591	44.420
IFN-Y	30.457	0	40.020	0	26.134	0
TNF-α	24.633	0	38.911	0	23.001	0
IL-4	25.510	0	31.383	2.850	16.628	0
IL-10	38.260	0	68.453	85.125	65.870	80.615

**Table 2:** Values of cytokines (in pg/mL) at different observation times expressed through the mean and median values in eyes operated with the LASIK method.

LASIK Participants	Tear Sampling Time					
Cytokine	t_0_		t_1_		t_2_	
	Mean	Median	Mean	Median	Mean	Median
IL-1β	9.611	0	16.595	4.460	13.370	2.270
IL-6	4.124	0	7.488	0	7.198	0
IL-8	10.909	0	31.993	39.970	33.645	41.150
IFN-Y	22.989	0	31.923	0	31.518	0
TNF-α	20.717	0	33.953	0	25.399	0
IL-4	24.478	0	31.529	6.310	26.373	0
IL-10	17.459	0	43.572	0	46.970	75.640

**Table 3:** Values of cytokines (in pg/mL) at different observation times expressed through the mean and median values in eyes operated with the PRK method.

PRK Participants	Tear Sampling Time					
Cytokine	t_0_		t_1_		t_2_	
	Mean	Median	Mean	Median	Mean	Median
IL-1β	11.260	1.120	21.862	1.880	9.057	0
IL-6	2.365	0	13.013	0	19.089	2.630
IL-8	15.494	0	31.689	41.710	42.734	47.860
IFN-Y	36.713	0	46.804	0	21.622	0
TNF-α	27.914	0	43.065	0	20.993	0
IL-4	26.375	4.170	31.261	2.070	8.464	0
IL-10	55.687	82.990	89.299	100.340	81.704	86.470

**Table 4:** Values of analyzed cytokines in two observation times, i.e., tear sampling, 1 h after intervention (t_1_LASIK and t_1_PRK) and 24 h after intervention (t_2_LASIK and t_2_PRK) along with a comparison between LASIK and PRK methods. In order to statistically process the data, the Mann–Whitney-U test was used.

	Cytokine													
Time	IL-1β		IL-6		IL-8		IFN-Y		TNF-α		IL-4		IL-10	
	Mean	*p* Value	Mean	*p* Value	Mean	*p* Value	Mean	*p* Value	Mean	*p* Value	Mean	*p* Value	Mean	*p* Value
t_1_LASIK	16.595	0.95	7.488	0.28	31.993	0.77	31.923	0.24	33.953	0.52	31.529	0.99	43.572	0.002
t_1_PRK	21.862		13.013		31.689		46.804		43.065		31.261		89.299	
t_2_LASIK	13.370	0.01	7.198	0.003	33.645	0.18	31.518	0.31	25.399	0.47	26.373	0.02	46.970	0.051
t_2_PRK	9.057		19.089		42.734		21.622		20.993		8.464		81.704	

**Table 5:** The table shows a positive correlation between pro-inflammatory and anti-inflammatory cytokines in the acute immune response after the LASIK method. Spearman’s correlation coefficient was used for statistical analysis.

	Tear Sampling Time	Spearman R	*p*-Value
IL-1β *correlatio* IL-4	t_1_LASIK	0.921	<0.001
	t_2_LASIK	0.906	<0.001
IL-1β *correlatio* IL-10	t_1_LASIK	0.604	<0.001
	t_2_LASIK	0.496	<0.01
IL-6 *correlatio* IL-4	t_1_LASIK	0.645	<0.001
	t_2_LASIK	0.571	<0.001
IL-6 *correlatio* IL-10	t_1_LASIK	0.638	<0.001
	t_2_LASIK	0.548	<0.01
IL-8 *correlatio* IL-4	t_1_LASIK	0.704	<0.001
	t_2_LASIK	0.525	<0.01
IL-8 *correlatio* IL-10	t_1_LASIK	0.559	<0.01
	t_2_LASIK	0.542	<0.01
IFN-γ *correlatio* IL-4	t_1_LASIK	0.857	<0.001
	t_2_LASIK	0.710	<0.001
IFN-γ *correlatio* IL-10	t_1_LASIK	0.690	<0.001
	t_2_LASIK	0.505	<0.01
TNF-α *correlatio* IL-4	t_1_LASIK	0.926	<0.001
	t_2_LASIK	0.846	<0.001
TNF-α *correlatio* IL-10	t_1_LASIK	0.724	<0.001
	t_2_LASIK	0.618	<0.001

**Table 6:** The table shows a positive correlation between pro-inflammatory cytokines and anti-inflammatory cytokines in the acute immune response after the PRK method. Spearman’s correlation coefficient was used for statistical analysis.

	Tear Sampling Time	Spearman R	*p*-Value
IL-1β *correlatio* IL-4	t_1_PRK	0.942	<0.001
	t_2_PRK	0.692	<0.001
IL-1β *correlatio* IL-10	t_1_PRK	0.588	<0.001
	t_2_PRK	0.223	>0.05
IL-6 *correlatio* IL-4	t_1_PRK	0.874	<0.001
	t_2_PRK	0.424	<0.01
IL-6 *correlatio* IL-10	t_1_PRK	0.703	<0.001
	t_2_PRK	0.303	>0.05
IL-8 *correlatio* IL-4	t_1_PRK	0.783	<0.001
	t_2_PRK	0.353	<0.05
IL-8 *correlatio* IL-10	t_1_PRK	0.465	<0.01
	t_2_PRK	0.104	>0.05
IFN-γ *correlatio* IL-4	t_1_PRK	0.819	<0.001
	t_2_PRK	0.404	<0.05
IFN-γ *correlatio* IL-10	t_1_PRK	0.534	<0.001
	t_2_PRK	0.171	>0.05
TNF-α *correlatio* IL-4	t_1_PRK	0.909	<0.001
	t_2_PRK	0.769	**<0.001**
TNF-α *correlatio* IL-10	t_1_PRK	0.650	**<0.001**
	t_2_PRK	0.542	**<0.001**

## 4. Discussion

In our study, the concentrations of all cytokines included in the test kit were determined by analyzing a certain number of tear fluid samples collected before excimer laser treatment (t_0_). The cytokines are TNF-α, TNF-β, IL-1β, IL-2, IL-4, IL-5, IL-6, IL-8, IL-10, IL-12, and IFN-γ. The samples were collected from the tears of healthy participants, which also served as the control group. In the LASIK and PRK group samples, IL-1β was detected in 35% and 54%, respectively. IL-6 was detected in 16% and in 30%, IL-8 in 26% and 35%, IFN-γ in 26% and 41%, TNF-α in 26% and 38%, IL-4 in 42% and 51%, and IL-10 in 19% and 59%, respectively. This led to the conclusion that the listed cytokines, including the ones with pro-inflammatory and anti-inflammatory effects, can be found in the tears of healthy people.

LaFrance et al. in their study compared different instruments and reagents to detect cytokines in the tears of healthy people. The instruments used in their study work on the principle of a “cytometric bead-based assay”. They obtained detectability rates in the following cytokines: IL-1β in 61%, IL-2 in 88%, IL-4 in 97%, IL-5 in 94%, IL-6 in 94%, IL-8 in 91%, IL-10 in 79%, IL-12 in 73%, IFN-γ in 91% and TNF-α in 12% of samples [22]. Carreno et al. in their study define the levels of different cytokines and chemokines in tears of healthy participants on the principle of a “multiplex bead-based assay”. They state that these levels can be used as a base value, i.e., as a control value to be compared with different cytokine levels in tears for various ocular diseases. In this study, in 100% of the samples, the following cytokines were detected: IL-1β, IL-2, IL-5, IL-6, IL-8 and IL-10. In contrast, IL-4 was detected in 22%, IL-12 in 6%, IFN-γ in 83%, and TNF-α in 50% of samples [23].

The production of biologically significant concentrations of pro-inflammatory cytokines in the tears of healthy people enables controlled activation (primarily of phagocytes) to remove impurities which impact the eye in daily activities while preventing uncontrolled inflammation, due to the presence of anti-inflammatory cytokines in tears, with the objective to preserve the transparency of optical media, primarily the cornea. This correlation is altered in the presence of ocular surface diseases, especially with an inflammatory type. It is interesting to see what happens immediately after performing excimer laser treatment, which was the aim of our study.

LASIK and PRK are the two most frequently performed refractive surgical procedures both globally and in our country. The corneal wound healing response is the determining factor for the safety and efficiency of these procedures. Clinical results, as well as numerous complications of these procedures, primarily haze, are directly linked to tissue repair processes and the unpredictable nature of the corneal cellular response. Both methods, apart from their positive impact in terms of correcting existing ametropia, lead to surgically induced trauma. The trauma response consists of a complex cascade of cellular interactions caused by cytokines, growth factors, and chemokines. An injury-related corneal response involves interlacing interactions of epithelial, stromal, neural, lacrimal cells, and immune system cells. The interactions between these cells determine the corneal response during wound healing and contribute to the regeneration and preservation of corneal anatomy and normal physiology. In PRK, excimer laser energy is directly applied on the de-epithelialized corneal stroma. During the wound healing response, the cornea may react with a more frequent occurrence of subepithelial blurring (haze) compared to LASIK. Namely, the main cause of haze is the interaction of cytokines between epithelium and stromal keratocytes, which activate keratocytes and biochemically degrades the stromal extra-cellular matrix. In the LASIK method, the excimer laser energy, after creating an intrastromal flap, is applied to the deeper layers of stroma, i.e., at a larger distance from the stimulatory effects of the corneal epithelium. The wound healing response after LASIK is characterized by a weaker interaction mediated by cytokines between the epithelium and stromal keratocytes compared to PRK, because in the LASIK procedure, the epithelial surface stays generally intact [4,24,25,26].

In our study, there were no postoperative complications like hypercorrection, hypocorrection, regression or subepithelial blurring (haze). The absence of complications is due to correct preoperative evaluation, regular postoperative follow-up of each patient, as well as following the surgical protocol, especially when performing the cooling PRK procedure [27]. The absence of haze is certainly the result of the regular implementation of prescribed postoperative therapy. Namely, after the PRK procedure, the patients used corticosteroid eyedrops (drops with dexamethasone 0.1% in the first month, and drops with prednisolone 0.5% in the second and third months). Kaji et al. in their study show that local application of corticosteroids (betamethasone) effectively suppresses the occurrence of haze after performing excimer laser keratectomy [28].

In our study, when analyzing Table 1, Table 2 and Table 3 and Figure 1, Figure 2 and Figure 3, it can be inferred that there is an increase in all cytokines, both pro-inflammatory and anti-inflammatory, in tears one hour after the intervention (t_1_) in relation to pre-operative values. When analyzing Table 1 and Figure 1, we can see that the values of IL-6 and IL-8 continue to rise until 24 h after the intervention (t_2_) in all examined eyes, while the values of IL-1β, IFN-γ, TNF-α, IL-4 and IL-10 reduce until 24 h after the procedure in all treated eyes. When analyzing Table 2 and Figure 2, we can see that the values of IL-6, IL-8, and IFN-γ continue to increase until 24 h after LASIK (t_2_), while the values of IL-1β, IL-4, TNF-α, and IL-10 reduce until 24 h after LASIK. When analyzing Table 3 and Figure 3, it can be seen that the values of IL-6 and IL-8 continue to rise until 24 h after PRK (t_2_), while the values of IL-1β, IFN-γ, TNF-α, IL-4, and IL-10 decrease until 24 h after PRK.

Since IL-6 and IL-8 are the only pro-inflammatory cytokines to maintain the dynamics of increase in values, when analyzing Table 4, we can see a statistical significance for values of IL-6 on the level *p* = 0.003 between t_2_LASIK and t_2_PRK, with significantly higher values of IL-6 in tears after PRK. When comparing the values of IL-8, there is no statistical significance between LASIK and PRK and one hour (t_1_) and 24 h (t_2_) post-operatively. The values of IL-1β as a pro-inflammatory cytokine show a statistically significant difference between LASIK and PRK 24 h post-operatively (t_2_) on the level *p* = 0.01, with higher values after LASIK. Similarly, IL-4 as an anti-inflammatory cytokine shows a difference in values between LASIK and PRK 24 h post-operatively (t_2_), with higher values after LASIK (*p* = 0.20). The values of IL-10 as an anti-inflammatory cytokine show statistically significant differences (*p* = 0.002) between LASIK and PRK one hour after treatment (t_1_) with higher values after PRK.

When analyzing the results in Table 5, we can see a positive correlation between all pro-inflammatory and anti-inflammatory cytokines after LASIK. Therefore, the increased value of every pro-inflammatory cytokine is followed by an increased value of every anti-inflammatory cytokine both 1 h (t_1_) and 24 h (t_2_) after the intervention. Hence, there is consistency between pro-inflammatory and anti-inflammatory response in the early postoperative period (the first 24 h) after LASIK.

When analyzing the results in Table 6, we can see a positive correlation between all pro-inflammatory and anti-inflammatory cytokines one hour post-operatively after PRK. This increased value of every pro-inflammatory cytokine is followed by an increased value of every anti-inflammatory cytokine in the given time of observation (t_1_). At 24 h after (t_2_) PRK, no correlation was observed between IL-1β and IL-10, IL-6 and IL-10, IL-8 and IL-10, and IFN-γ and IL-10. In contrast, for other correlations between pro-inflammatory and anti-inflammatory cytokines in the same observation time (24 h after, t_2_), a positive correlation was observed. Since IL-10 shows higher values after PRK than after LASIK one hour after the intervention (t_1_), we can conclude that IL-10 displays its anti-inflammatory effect sooner than IL-4.

Leonardi A et al. in their study [29] examined the level of different cytokines and chemokines in the tears of myopic eyes before and after LASIK intervention, and in corneal fibroblast cultures before and after excimer laser treatment. Tears were sampled from the eyes of 15 myopic patients before, 1 h after, and 24 h after LASIK intervention. Cytokine values in tears were determined based on the principle of multiplex bead analysis. For IL-1β, they obtained an increase in the level of this cytokine in 6 patients (40% of samples) 1 h after LASIK intervention. They detected IL-4 in tear samples before LASIK treatment, but at a concentration below the cutoff of 5 pg/mL in 11 out of a total of 15 patients. After LASIK treatment, there were no changes in IL-4 concentration in tear samples. Furthermore, before LASIK treatment, IL-6 was not detected in the tears of patients. Postoperatively, 24 h after LASIK, in 9 out of a total of 15 patients (60%), there was an increase in the level of IL-6 in the tear samples. The only cytokine present in all pre-LASIK tear samples in this study was IL-8. Compared to pre-treatment values, in 8 out of 15 patients (53%), there was an increase in IL-8 levels in tear samples 24 h after treatment. Furthermore, IL-10 was not detected in tear samples either before or after LASIK treatment. Leonardi et al. detect IFN-γ in tear samples before LASIK treatment but in a concentration that is below the limit of 5 pg/mL in 11 out of a total of 15 patients. After LASIK treatment, there were no changes in the concentration of this cytokine in the tear samples. Finally, in the study, the level of TNF-α in tear samples 1 h after LASIK intervention was increased in 7 out of a total of 15 patients (47%). Leonardi et al. concluded that after excimer laser treatment cytokines are released to modulate the wound-healing process; however, they can potentially induce inflammation.

Alio JL. and Javaloy J. in their review article [10] conclude that adequate management of postoperative inflammation is essential after photorefractive procedures. Successful modulation of the biochemical inflammatory process and cellular wound healing response seems to be important after surface procedures to achieve an excellent visual outcome.

One of the limitations of our study is that, mainly because of the range in refractive errors, there were no postoperative complications such as corneal haze; therefore, we were not able to examine if and how the inconsistency between the pro- and anti-inflammatory response in the early postoperative period can be directly correlated to the appearance of these adverse events. Due to lack of a long-term follow up in this study, we were also not able to correlate these findings with potential long-term issues such as ocular surface symptoms and dry eye parameters. We assume that future research may conclude that a specific imbalance between the pro- and anti-inflammatory response in favor of the values of pro-inflammatory cytokines in tears, in the early postoperative period, can be used as a biomarker for these complications. Furthermore, since both eyes from most participants were included in the analysis, intra-subject inter-eye correlation could not be completely excluded. Therefore, the statistical independence of all observations cannot be assumed, which represents a limitation of the present study.

## 5. Conclusions

By integrating all previously analyzed results, our study concludes that there is consistency between the inflammatory and anti-inflammatory response of the ocular surface in the early postoperative period, which persists longer after LASIK compared to PRK.

## Data Availability

The data presented in this study are available on request from the first and the corresponding author; the datasets are archived in the clinics where participants were treated. The data are not publicly available as they contain information that could compromise the privacy of the participants.
